# Can Mating Disruption Be a Possible Route to Control Plum Fruit Moth in Mediterranean Environments?

**DOI:** 10.3390/insects11090589

**Published:** 2020-09-01

**Authors:** Gabriella Lo Verde, Salvatore Guarino, Stefano Barone, Roberto Rizzo

**Affiliations:** 1 Department of Agricultural, Food and Forest Sciences, University of Palermo Viale delle Scienze, 90128 Palermo, Italy; gabriella.loverde@unipa.it (G.L.V.); stefano.barone@unipa.it (S.B.); 2 Institute of Biosciences and Bioresources (IBBR), National Research Council of Italy (CNR), Corso Calatafimi 414, 90129 Palermo, Italy; salvatore.guarino@ibbr.cnr.it; 3 CREA Research Centre for Plant Protection and Certification, SS.113, Km 245,5, 90011 Bagheria, Palermo, Italy

**Keywords:** *Grapholita funebrana*, Tortricidae, sex pheromones, integrated pest management

## Abstract

**Simple Summary:**

*Grapholita funebrana* is a main pest of plum throughout the Palearctic region. The management of this pest is generally carried out with chemical insecticides. In this study we investigated the suitability of the mating disruption as alternative method of control of this pest. Experiments were carried out in organic plum orchards during 2012 and 2014. Trap catches and fruit sampling were carried out to estimate the efficacy of this technique in reducing males catch and fruit infestation. The results indicated that the males caught in traps placed in the treatment plots was always significantly lower than untreated plots. The chemical analysis of the pheromone emission from the dispenser, carried out by solid-phase micro-extraction followed by gas chromatography, indicated an optimal duration of these tool for at least 60 days of field exposure. Fruit sampling evidenced that pheromone treatment significantly reduced fruit infestation, but not economic damage, particularly on the cultivar for which a high susceptibility to the moth infestation is known.

**Abstract:**

Control of the plum fruit moth, *Grapholita funebrana* Treitschke (Lepidoptera: Tortricidae), has been mainly based on the use of chemical insecticides, which can cause undesirable side effects, leading to a growing interest towards alternative sustainable strategies. The aim of this study was to evaluate the effect of the mating disruption technique on *G. funebrana* infestation in plum orchards, by comparing the number of male captures in pheromone-baited traps, and evaluating the damage to fruits in plots treated with the pheromone dispersers and in control plots. The study was carried out in 2012 and 2014 in three organic plum orchards, on the cultivars Angeleno, Friar, President and Stanley. To evaluate the pheromone emission curve of the dispensers from the openings to the end of the trials, a chemical analysis was carried out by solid phase micro-extraction followed by gas chromatography, followed by mass spectrometry. In all years and orchards the mean number of males caught in traps placed in the treatment plots was always significantly lower than untreated plots. Pheromone emission from the dispensers was highest at the opening, and was still considerable at 54 days of field exposure, while it significantly decreased after 72 days of field exposure. Cultivar was confirmed to be an essential factor in determining the fruit infestation level. Pheromone treatment significantly reduced fruit infestation, but not economic damage.

## 1. Introduction

The growing demand for plums is due to their nutritional peculiarities, as they seem to provide a variety of health benefits, thanks to their healthful compounds [1,2,3,4]. As a consequence, there has been a worldwide increase in plum cultivated areas (*Prunus domestica* L. and *Prunus salicina* Lindl.), reaching 2.7 million hectares in 2018 [5]. The global production of plums increased at an average annual rate of 2.3% in the period 2007–2018, and reached the peak of plum fruits production in 2018, with 12.6 million tons [5].

*Grapholita funebrana* Treitschke (Lepidoptera Tortricidae), commonly called the plum fruit moth (PFM), is an oligophagous species, feeding on the fruits of several hosts within the family Rosaceae [6,7,8]. The species is considered the main pest of plums throughout the Palearctic region [9].

The number of PFM generations per year varies depending on climate: in warmer areas, as in central and southern Italy, three generations per year can occur [8,10,11]. Females of PFM lay eggs on the exocarp surface of developing fruits of host plants [12]. Neonate larvae bore into fruits, where they feed and develop. Damage is due to the feeding activity of the larvae inside the fruits, leading to changes in fruit coloration, early ripening and fruit fall, with consequent yield decreases [8,10]. Furthermore, infested fruits show penetration holes (characterized by the presence of gum) made by neonate larvae, and exit holes made by mature larvae leaving the fruit [13]. The losses of production of plums determined by PFM infestations can be quite significant, especially in plum cultivars particularly susceptible to this pest [8,11].

The control of PFM in plum orchards has been mainly based on the use of chemical insecticides, which can cause side effects on beneficial insects, both pollinators and natural enemies, and can have serious implications for human health and the environment [14], or the development of resistance, as found in other tortricids, like *Grapholita molesta* Busck [15,16], *Cydia pomonella* L. [17,18,19] and *Lobesia botrana* (Denis & Schiffermuller) [20]. Moreover, in organic plum orchards, moth control is particularly difficult due to the small number of authorised products.

In this context, also in consideration that the European Union policy (Directive 2009/128/CE) is encouraging a reduction in the use of pesticides [21], growing research attention has been devoted to the development of alternative environmentally friendly and sustainable strategies to control insect pests of agricultural importance [22,23,24,25,26,27]. Among them, the manipulation of insect behavior with the use of pheromones is receiving increasing attention [28,29,30,31]. Pheromones have been effectively applied in the management of dangerous lepidopteran species, through the technique of the mating disruption, based on the release of high amounts of synthetic sex pheromones into a crop, thus interfering with the mate finding processes of a given pest species [32,33,34,35].

In the case of PFM, the sex pheromone is characterized by two main active compounds, i.e., (*Z*)-8-dodecenyl acetate and (*E*)-8-dodecenyl acetate [36], with (*Z*)-8-dodecen-1-ol as a minor component [37]. The sexual behavior exhibited by PFM is similar to other tortricid pest species that are controlled by mating disruption, thus suggesting the possibility of the application of this technique for PFM too. To date, despite the high economic importance of this insect pest, limited information is available about the application of mating disruption for its control [38,39]. The objective of this research was to evaluate the efficacy of the mating disruption technique in Sicilian organic plum orchards. The effect of the mating disruption method on PFM population and fruit damage was assessed by comparing the captures of PFM males in pheromone-baited traps and evaluating the infestation of fruits of four different plum cultivars in plots treated with the pheromone and in control plots.

## 2. Materials and Methods

### 2.1. Study Areas and Dispenser Placement

The research was performed in three different Sicilian organic plum orchards located in San Giuseppe Jato, Palermo province, Italy, in two different years. In 2012, field trials were carried out in a plum orchard of 3 hectares (37°99′87″ N, 13°21′12″ E), in which pheromone dispensers were placed (treated plot), while an untreated plum orchard of 1 ha, about 500 m away from the first one, was used as the control plot. In 2014, trials were carried out in two different orchards. In the first one (Orchard A, 37°99′31″ N, 13°22′47″ E), the pheromone dispensers were placed in 4 ha, while a surface of 1 ha, about 400 m far from it, was used as the control plot. In the second one, dispensers were placed in a 3 ha plum orchard (Orchard B, 37°99′57″ N, 13°20′74″ E), and a surface of 1 ha, about 200 m away, was used as the control.

All the orchards were on flat land and regularly shaped, planted with 8-years-old plum trees grafted on Mirabolano (*Prunus cerasifera* Ehrh.) rootstocks trained to a vase shape. Plum trees were spaced with 6 m between rows and 3 m between trees within a row. The four cultivars chosen for the study were Angeleno and Friar (*Prunus salicina* Lindl.) and Stanley and President (*Prunus domestica* L.). In all plum orchards, the four cultivars were distributed along eight rows (two rows for each cultivar). This distribution pattern of the cultivars was repeated over the entire surface of each orchard. The trees were managed using routine organic cultural practices; during the research, no insecticide treatments were carried out in the orchards.

Isomate^®^ OFM Rosso Flex (Shin-Etsu Chemical Co. Ltd., Ohtemachi Chiyoda-ku, Tokyo, Japan) pheromone dispensers were placed in the field once during the season (on 30 March in all years), before the adult of PFM of the wintering generation emerged. Dispensers were hung in the upper third of tree canopy with a density of 550 pieces per hectare. Two dispensers per tree were placed in the outer row that delimited each experimental plot, while one dispenser per tree was placed in the rows following the latter and one dispenser every two trees within its perimeter. According to the manufacturer, each dispenser was loaded with 254 mg of pheromone mixture. Pheromone dispensers consisted of two parallel capillary tubes made of polyethylene sealed at the ends, filled with the PFM pheromone blend, consisting of (*Z*)-8-dodecenyl acetate (89.6%), (*E*)-8-dodecenyl acetate (5.4%) and (*Z*)-8-dodecen-1-ol (1%). The gap in the middle allows each dispenser to form a loop that can be easily deployed by placing the dispenser on a branch.

### 2.2. Trap Captures

The monitoring of PFM flight was carried out by placing three pheromone-baited (Isagro, Milano, Italy) sticky traps in each of the six experimental plots from the second half of March. Traps were checked every 10 days. Male genitalia extraction and observation of insects caught at each date were done to confirm the specific identification of the tortricid species.

### 2.3. Fruit Infestation

To compare the PFM field infestation occurring in the different cultivars present in the pheromone treated plots and in the control plots, fruit samplings were carried out, followed by dissection under a microscope to assess the presence of larvae. In detail, in 2012, for each cultivar, four groups of three plum trees, randomly chosen in each plot, were used for fruit sampling, while in 2014, three groups of three plum trees were used for each orchard and each cultivar. On these trees, starting from the first catches in the traps (on 9 April in 2012 and on 5 April in 2014), field observations were carried out on 100 fruits from each cultivar in each experimental treatment plot, in order to detect the first eggs laid by PFM. Afterwards, fruit sampling was done every two weeks, from 28 May to 24 July 2012, and from 23 May to 30 July 2014. In 2012, at each sampling date, 8 fruits per tree (96 per cultivar) were collected randomly around the canopy, whereas in 2014, 10 fruits per tree (90 per cultivar) were collected. In 2012, the cultivar Friar was not sampled due to inadequate fruit production. All fruits were then kept in the Department of Agricultural, Food, and Forest Science (University of Palermo, Palermo, Italy), and were dissected under a stereomicroscope to record the presence of PFM larvae. Fruit was considered infested when larvae or their penetration and exit holes were present.

### 2.4. Estimation of Pheromone Release from Dispensers

In 2014, the residual emission of sex pheromone by field dispensers periodically collected at each fruit sampling was evaluated, in comparison with a new dispenser. Three dispensers were periodically collected at each fruit sampling, extracted and analyzed by GC-MS. The pheromone emission rate from the dispenser was analyzed by headspace using a solid phase micro-extraction (SPME) in static air [40], an equilibrium process involving the headspace and the polymeric fiber stationary phase [41]. The stationary phase used as the coatings was poly(dimethylsiloxane) (PDMS, 100 μm). A manual SPME holder from the same manufacturer was used for injections. Fibers were conditioned in a gas chromatograph injector port as recommended by the manufacturer: PDMS at 250 °C for 30 min. SPME extractions were performed in climatic chambers (27 ± 2 °C and 50 ± 5% RH). For pheromone collection, the releasers’ samples were placed into 40 mL vials, which were sealed with a poly(tetrafluoroethylene) silicon septum-lined cap (Supelco, Bellefonte, PA, USA). An SPME needle was then inserted through the septum and headspace volatiles were absorbed on the exposed fiber for 30 min in a conditioned room (29 ± 1 °C; 40 ± 5% RH). The release rates of the dispensers were measured at the opening and after 54, 72, 89, and 122 days of field exposure. Experiments were replicated three times for each day of sampling from the releaser opening and field exposure. In order to perform a chemical analysis on the collected pheromone, immediately after the end of the sampling time, the loaded fiber was desorbed in the gas chromatograph inlet port for 2 min. Coupled gas chromatography-mass spectrometry (GC-MS) analyses of the headspace extracts from the pheromone releasers were performed on an Agilent 6890 GC system interfaced with an MS5973 quadruple mass spectrometer, which was injected onto a DB5-MS column in 1/50 split mode. Injector and detector temperatures were 260 °C and 280 °C respectively. Helium was used as the carrier gas. The GC oven temperature was set at 40 °C for 5 min, and then increased by 10 °C/min to 250 °C. Electron impact ionization spectra were obtained at 70 eV, recording mass spectra from 40 to 550 amu. The measurements of the pheromone emission rate were accomplished by integrating the pheromones’ peaks.

### 2.5. Statistical Analysis

Data on the number of males of PFM caught in the traps were analyzed, after a root square transformation, using a general linear model (GLM) procedure, in which the factors’ treatment (pheromone treated plot/control plot), date and their interaction were included. Tukey’s HSD test (*p* < 0.05) was then applied to assess the significant difference between treatments.

As infestation data were recorded as the presence/absence on fruits, a binary logistic regression analysis was performed, separately for each year and orchard, to assess the significance of the independent factors. Treatment (placement of pheromone dispenser vs. control), cultivar and date were included in the models as factors. Moreover, as variable susceptibility of the studied cultivars was known from a previous study [8], the interaction effect between the treatment and cultivar was included in the analysis.

The mean of the chromatographic areas obtained from headspace SPME pheromone collection of releaser with different field age were integrated and compared by using one-way ANOVA, followed by Tukey’s HSD test (*p* < 0.05).

MINITAB software was used for all statistical analyses (Minitab Inc., State College, PA, USA).

## 3. Results

### 3.1. Trap Captures

**Figure 1:**
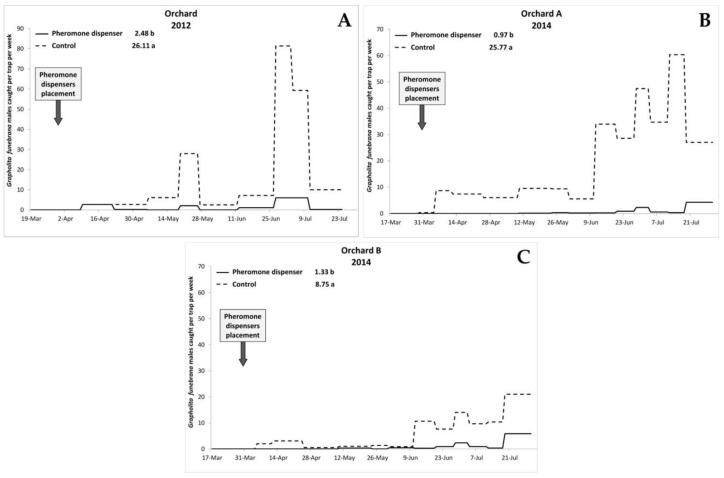
Trend of *Grapholita funebrana* males catches per trap per week in (**A**) Orchard 2012, (**B**) Orchard A 2014 and (**C**) Orchard B 2014. The mean number of captures per trap over the entire study period is reported in the legend; different letters indicate significant differences between the means (GLM followed by Tukey’s HSD test, *p* < 0.05).

Overall, 2246 males were caught in the traps, of which 2214 were *G. funebrana*, while 32 were belonging to *G. molesta*. Figure 1 shows the trend of PFM male captures per trap per week. First catches in the traps were recorded on 9 April in 2012 and on 5 April in 2014, in both Orchards A and B. An increase in catches was recorded from the second half of April, corresponding to the first PFM generation. After this date, in all years, the number of males in the traps was clearly higher in the control plots (Figure 1). In 2012, the highest number of captures was recorded on 27 June, with a mean number of 81.3 males per trap. In 2014, in Orchard A, the peak was recorded on 13 July (60.3 males per trap); in Orchard B it was recorded on 19 July (21 males per trap). In pheromone plots, the maximum number of males in the traps were recorded on 27 June 2012 (6 males per trap), and on 19 July 2014 (4.3 males per trap) in Orchard A, and on the same date in Orchard B (5.8 males per trap). Statistical analysis showed that the effect of treatment, date and their interaction were significant in all years (Table 1). The average number of males caught in traps placed in the treatment plots was always significantly lower than in untreated plots (Figure 1). Moreover, treatment was the most important of the factors included in the analyses (Table 1). Overall, the GLM models allowed us to effectively explain the variability in the caught number of PFM males, as shown by the values of adjusted R^2^ values: 80.50 in 2012 and 92.40 in 2014 for Orchard A and 78.97 in 2014 for Orchard B.

### 3.2. Fruits Infestation

**Figure 2:**
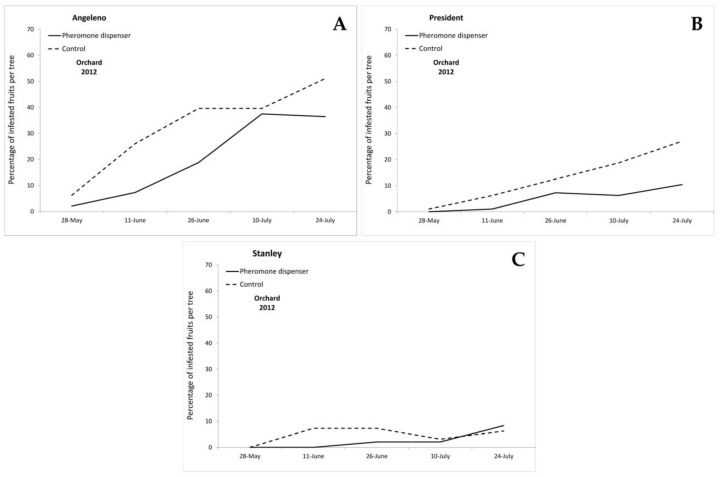
Infestation trend (mean percentage of infested fruits per tree) recorded in 2012 in the cultivars (**A**) Angeleno, (**B**) President and (**C**) Stanley.

**Figure 3:**
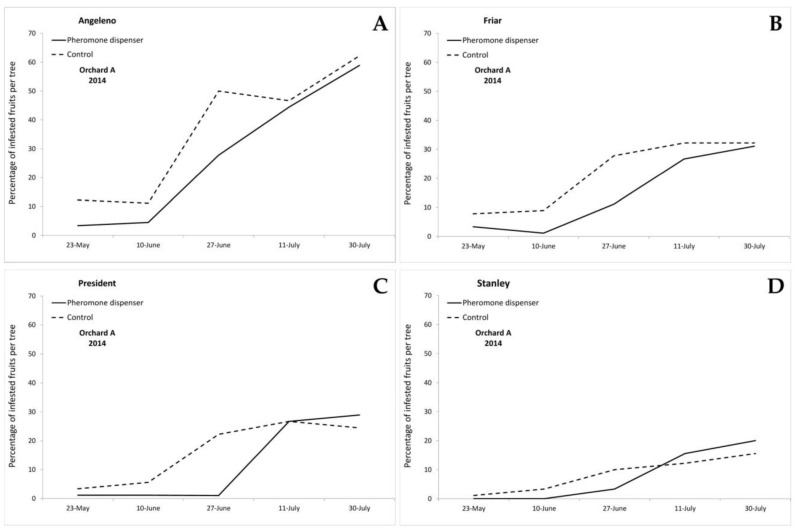
Infestation trend (mean percentage of infested fruits per tree) recorded in 2014 in Orchard A in the cultivars (**A**) Angeleno, (**B**) Friar, (**C**) President, and (**D**) Stanley.

**Figure 4:**
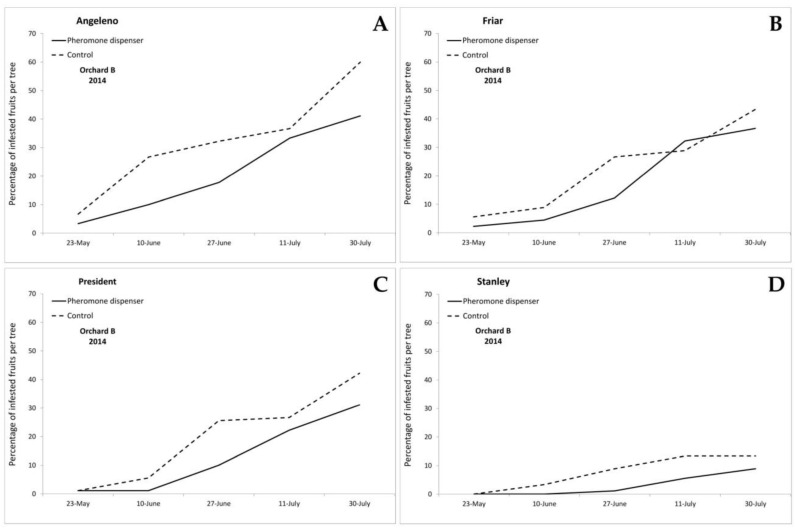
Infestation trend (mean percentage of infested fruits per tree) recorded in 2014 in Orchard B in the cultivars (**A**) Angeleno, (**B**) Friar, (**C**) President, and (**D**) Stanley.

The first eggs on plums were found on 18 May 2012 and on 9 May 2014 in both orchards. In 2012, the first infested fruits were recorded on 28 May 2012 on Angeleno fruits, in both treated and control plots, and on President on fruits sampled in the control plot, whereas the first infested fruits on the Stanley cultivar were found on 11 June in the control plot (Figure 2). In 2014, in Orchard A, the first infested fruits were recorded on 23 May in all cultivars and treatments, with the only exception of the treated plot of the Stanley cultivar, in which infested fruits were recorded starting from 27 June (Figure 3). In Orchard B, the first infested fruits were recorded on 23 May, in both treated and control plots of Angeleno, Friar and President, while in the Stanley cultivar, infested fruits were recorded starting from 10 June in the control plot and from 27 June in the pheromone treated plot (Figure 4). During all years and orchards, on most of the sampling dates, the mean number of infested fruits per tree was lower in the treated plots. Angeleno was always the most infested cultivar and the only one in which a lower number of infested fruits per tree was recorded in the treated plot for all years, orchards and sampling dates (Figure 2, Figure 3 and Figure 4).

Statistical analysis showed in all years and orchards a significant effect of the factors treatment, cultivar and date (Table 2). The interaction between cultivar and treatment was not significant, indicating that the effect of the treatment does not depend on the cultivar, and at the same time that the differences among the cultivars are not due to the treatment. Overall, the values of the adjusted R^2^ values obtained in the GLM models allowed us to explain 56.15%, 71.11% and 71.02% of the variability in the infestation found in 2012 and in 2014 in Orchard A, and in 2014 in Orchard B, respectively. Moreover, the most important among the factors included in the analyses was the date, always followed by the cultivar (Table 2).

However, the infestation percentages found on the last sampling date (corresponding to fruit harvesting) showed a reduction, due to mating disruption in treated plots of Angeleno and Friar in all years (Table 3). In the President cultivar, a reduced infestation due to mating disruption was found only in 2012 and 2014 in Orchard B, while for Stanley, a reduction was observed only in 2014 in Orchard B. For Angeleno, the most susceptible cultivar [8], the infestation in treated plots was quite high in all years (Table 3). In Orchard A, no differences were recorded between treatment and control with regard to fruit infestation.

### 3.3. Estimation of Pheromone Release from Dispensers

**Figure 5:**
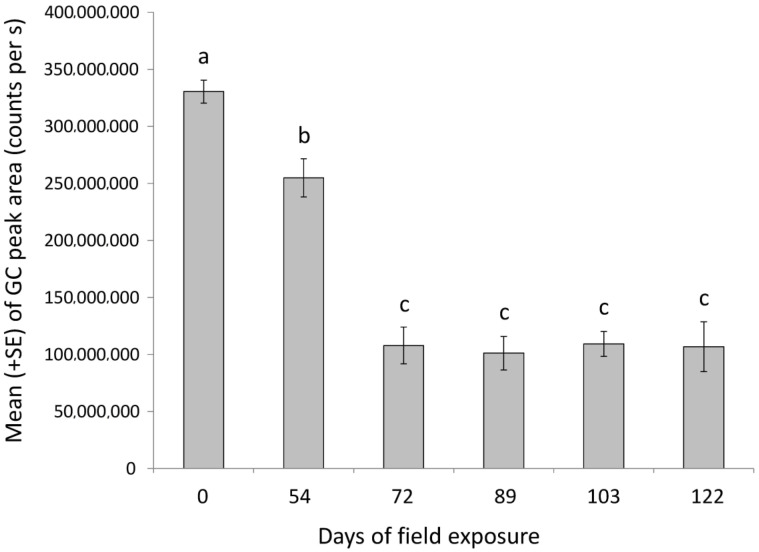
Mean (+SE) release rates of *Grapholyta funebrana* pheromone from dispensers measured by collecting the volatiles, by using the SPME headspace method at 22 °C. No letter in common indicates significant differences for *p* < 0.05 (ANOVA followed by Tukey’s HSD test).

The mean release rates of pheromone from dispensers, measured by SPME in static air, are shown in Figure 5. The pheromone emission significantly decreased over the field-ageing period for both dispensers placed in the field (*F* = 46.09; *df* = 5; *p* < 0.001; ANOVA). Pheromone emission was higher at releaser opening than at all the other times of sampling (*p* < 0.05, ANOVA followed by Tukey’s test). Releasers with 54 days of field exposure emitted more pheromone emission than the releasers with a longer duration of field exposure (*p* < 0.01; ANOVA followed Tukey’s HSD test). However, no statistical differences were observed in pheromone emission from releasers recovered from 72 to 122 days of field exposure.

## 4. Discussion

Pheromone-mediated mating disruption aims to interfere with mate finding, reduce insect population growth, and prevent crop damage [42]. The main system for mate finding in moths is due to the female release of small amounts of sex pheromone, which is detected by males through their highly sensitive neurosensory structures [43,44]. In addition to the prevention of mating, pheromone treatment can also result in a delay in mating, thus impacting the fitness and subsequent population dynamics of the target insect pest [42]. Mating disruption using pheromone dispensers, distributed into a crop, has had great success for the control of harmful tortricids like *L. botrana* [45,46,47], *G. molesta* [48,49] and *C. pomonella* [50,51]. Nevertheless, some difficulties can occur when high populations of pests are present, and it may be necessary to reduce the population of the first moth generation, as found for PFM by [52]. For this reason, mating disruption should be considered as one control method within a structured strategy of integrated pest management, including other control methods [52,53]. With regard to PFM, few studies on its control by pheromone-based methods have been carried out in central Europe [39,52,53,54]. In Italy, the effect of pheromone-based methods on PFM was assessed in studies carried out in central and northern Italian regions on the Stanley cultivar, adopting the false-trail or the mating disruption methods [6,9]; no information is available for plum orchards in Mediterranean environments.

Our results show that catches of male PFM moths in pheromone traps were strongly reduced in the treated plots in comparison with the control plots, as found in other studies [9,49,53]. In the control plots, the trend of PFM males catches clearly shows the occurrence of peaks corresponding to at least two different PFM generations in the study period. Despite the reduction in male captures, the population trend in treated plots showed capture peaks on the same dates as the control plot. The pheromone emission rate from the dispensers significantly decreased with field aging until day 72; specifically, the emission of pheromone from the dispenser after opening decreased by about 25% on the 54th day of field exposure, and by 66% on the 72nd day of field exposure after opening. Afterwards, the pheromone emission was stable. The lower levels of male catches during the entire sampling period in treated plots led us to surmise that the pheromone, despite a lowering release rate, was still effective in reducing the captures of PFM males until the end of July (about 120 days after dispenser placement in the field), when the insect population increased to its highest levels. However, the pheromone emission results also suggest that a second application of pheromone dispensers or the use of automatic aerosol devices releasing pheromone puffs at programmed time intervals [31,46] might give a better effect on PFM mating disruption.

Fruit infestation recorded in control plots was higher compared to pheromone treated plots in the entire sampling period in all cultivars and years, with the only exceptions being samples of the cultivars Friar (Orchard B) and Stanley (Orchard A) collected on 11 July 2014. It should be noted that, in control plots, the infestation increase followed the trend of catches in the traps, which is related to an increase of the insect population due to the development of the second generation. In the pheromone treated plots, the reduction in trap catches was more evident than the reduction in the fruit infestation level. This led us to suppose that, despite the fact that the pheromone level in the field was effective in reducing trap attractiveness for PFM males, it did not inhibit the insect mating and oviposition to the same extent. This was particularly evident in Orchard A, in which no differences in the fruit infestation were found at harvest, despite the very low PFM catches recorded in the pheromone treated plot. Moreover, in Orchard B, the infestation level in both treated and control plots was higher than in 2012, although the number of PFM males in traps in the control plot was very low in 2014 compared to 2012. This suggests that the use of pheromone traps to evaluate the efficacy of the mating disruption technique can give information about changes in moth flights, but cannot be considered reliable as a stand-alone monitoring tool in areas treated with mating disruption. This has been observed also in other studies carried out on tortricid moth control in apple orchards and vineyards in Italy, as the information provided by traps needs frequent field scouting to evaluate the effective control of target species through mating disruption [46].

In our study, the reduction in fruit infestation recorded in treated plots compared to control plots cannot be considered suitable from an economic point of view, particularly in the most susceptible cultivar, Angeleno, in which the infestation in treated plots was 36% (2012), 59% (Orchard A) and 41% (Orchard B). In contrast, other studies carried out in central Europe showed that the application of mating disruption significantly reduced fruit damage and maintained a percentage of infested fruits below the economic injury level [49].

Our study confirmed that cultivar represents an important factor in determining the fruit infestation level, as already demonstrated by [8]. In particular, Stanley, a cultivar previously studied in Italy by [6,9], was less infested than Angeleno, Friar and President [8,11]. This complicates the comparison between our results and most of the literature, in which the cultivar is not reported.

The probability of fruit infestation is related both to the cultivar and to the density of the insect pest population. The PFM population density can be affected by environmental factors, such as the occurrence of wild host plants, like *Prunus spinosa* L. [9,52]. It could be useful to lower the density of the PFM generation, by chemical or biological methods [52,55], like the use of entomopathogenic nematodes against diapausing larvae [53].

## 5. Conclusions

This study showed that mating disruption application in the management of PFM can reduce the population level and the fruit infestation level. However, the use of mating disruption as a stand-alone method for controlling the PFM in Mediterranean plum orchards did not prove to be sufficient to contain the infestation below the economic damage threshold, especially on cultivars that are particularly susceptible to the PFM attack and when a high population density of the insect occurs. Therefore, it will require supplemental control methods to reduce fruit infestation. From this perspective, accurate economic assessments relating to possible combinations of control methods should be carried out, in order to achieve a sustainable use of pesticides, as promoted by the European Union (Directive 2009/128/CE). However, using the mating disruption method requires know-how and experience, while at present, just a few publications are available for PFM compared to other tortricids like *L. botrana* or *G. molesta*.

Interesting issues to be investigated in future studies could be aimed at improving knowledge about the susceptibility of the most widely cultivated cultivars, which is an essential factor in determining the fruit infestation level. Moreover, more efforts should be carried out to assess the effectiveness of the mating disruption method in the same orchards in consecutive years, to evaluate the additive effect on the reduction of the population. However, it should be useful to optimise the emission of an adequate amount of pheromone in the field that can provide control of PFM throughout the entire season, for example, through the use of automatic aerosol devices [31,46]. Furthermore, improved knowledge about distribution and forecast models [56,57] could provide further opportunities for the integrated management of this key pest in plum orchards.

## Figures and Tables

**Table 1:** Results of the general linear model (GLM) analyses, performed each year separately on the number of *Grepholita funebrana* males caught in the traps, and effect of the different factors considered (asterisks indicate significant factors within each year, * *p* < 0.01).

Factors	Orchard 2012		Orchard A 2014		Orchard B 2014	
	*df*	*F*-Values	*df*	*F*-Values	*df*	*F*-Values
Treatment	1	101.41 *	1	620.15 *	1	110.91 *
Date	8	12.16 *	12	18.73 *	11	13.55 *
Treatment * Date	8	4.64 *	12	9.65 *	11	2.70 *
Error	36		52		48	
Total	53		77		71	

**Table 2:** Results of the binary logistic regression, separately performed for each year and orchard, on the infestation data and effect of the different factors considered (asterisks indicate significant factors within each year, * *p* < 0.01). All of the goodness-of-fit tests of the reported models were found to be non-significant.

Factors	Orchard 2012		Orchard A 2014		Orchard B 2014	
	*df*	Chi-Squared	*df*	Chi-Squared	*df*	Chi-Squared
Regression	9	470.642 *	11	635.21 *	11	597.45 *
Treatment	1	20.42 *	1	9.54 *	1	17.39 *
Cultivar	2	107.49 *	3	77.21 *	3	89.35 *
Date	4	190.87 *	4	436.95 *	4	391.02 *
Treatment * Cultivar	2	1.83	3	2.96	3	3.60
Error	350		348		348	
Total	359		359		359	

**Table 3:** Percentage of infestation recorded at harvest, and differences between treated and control in the different cultivars and years.

	Orchard 2012			Orchard A 2014			Orchard B 2014		
Cultivar	Pheromone Dispenser	Control	Difference	Pheromone Dispenser	Control	Difference	Pheromone Dispenser	Control	Difference
Angeleno	36.46	51.04	−14.58	58.89	62.22	−3.33	41.11	60	−18.89
Friar	-	-	-	31.11	32.22	−1.11	36.67	43.33	−6.66
President	10.42	27.08	−16.66	28.89	24.44	+4.45	31.11	42.22	−11.11
Stanley	8.33	6.25	+2.08	20.00	15.56	+4.44	8.89	13.33	−4.44
